# Detection of JAK2 V617F mutation increases the diagnosis of myeloproliferative neoplasms

**DOI:** 10.3892/ol.2014.2801

**Published:** 2014-12-12

**Authors:** SHU-PENG ZHANG, HUI LI, REN-SHENG LAI

**Affiliations:** Department of Pathology, The Affiliated Hospital of Nanjing University of Traditional Chinese Medicine, Nanjing, Jiangsu 210029, P.R. China

**Keywords:** Janus kinase 2 gene, V617F, gene test, direct sequencing, myeloproliferative neoplasms

## Abstract

The Janus kinase (JAK)2 gene, which is located on chromosome 9p24, is involved in the signaling transduction pathways of the hematopoietic and immune system. Mutations in the JAK2 gene have served as disease markers for myeloproliferative neoplasms (MPNs). The aim of the present study was to investigate the occurrence of the JAK2 gene mutation in 140 clinical samples, and to evaluate its clinical significance in MPNs and other hematological diseases. Genomic DNA was extracted from the peripheral blood leukocytes or bone marrow karyocytes of 140 clinical samples, which included 130 patients with various types of hematological disease and 10 control patients. In addition, exons 12 and 14 of the JAK2 gene were analyzed by direct sequencing and the mutation rates of various MPN subtypes were evaluated. Of the 140 samples, exons 12 and 14 were tested in 74 samples, however, exon 14 only was tested in 66 samples. No mutations were identified in exon 12. The V617F mutation rate in polycythemia vera was 82.1% (23/28), and the mutation rates in essential thrombocythemia histiocytosis, primary myelofibrosis and other MPNs were 53.1% (17/32), 40.0% (4/10) and 60.0% (6/10), respectively. Therefore, the total mutation rate of the JAK2 gene in MPN was 62.5% (50/80). For non-MPN hematological diseases, four V617F mutations were detected in samples of leukocytosis of unknown origin (4/12), however, no JAK2 V617F mutations were identified in the 10 controls. Therefore, JAK2 V617F mutations may present a novel marker for diagnosis of MPNs. Furthermore, the direct sequencing method appeared to be satisfactory for the clinical gene testing of hematological samples.

## Introduction

Janus kinase (JAK) is a non-receptor tyrosine kinase, and the JAK family is comprised of JAK1, JAK2, JAK3 and tyrosine kinase 2. The JAK gene consists of four sections: An N-terminal consisting of a protein 4.1R, ezrin, radixin, moesin domain, which interacts with cytokine receptors, a Src homology 2 (SH2) domain, a pseudokinase domain (JH2) adjacent to the SH2 domain and a C-terminal kinase domain (JH1) (1). JAK2 is an important member of the JAK family, which is located on chromosome 9p24, and its structure is highly homologous with other members of the JAK family. JAK2 is widely distributed in the cytoplasm of somatic cells, and is involved in signaling transduction pathways of the hematopoietic and immune system. Epidermal growth factor, platelet derived growth factor, colony stimulating factor, interleukin 3 and erythropoietin mediate cell proliferation, differentiation and apoptosis via the JAK2 signal transduction pathway. It has been demonstrated that the JAK2 V617F point mutation, a common molecular genetic abnormality, occurs in polycythemia vera (PV), essential thrombocythemia histiocytosis (ET) and primary myelofibrosis (PMF); therefore, the JAK2 mutation may be an important diagnostic tool for the detection of myeloproliferative neoplasms (MPNs) (2). The mutation occurs at base position 1849 in exon 14; a homozygous G to T transversion occurs, which causes phenylalanine to be substituted for valine at position 617 of JAK2 (V617F), consequently increasing the tyrosine kinase activity (3–8). It has been determined that the JAK2 V617F mutation occurs at the stem cell level, and is the major molecular mechanism, as well as a potential diagnostic marker, for the development of MPNs, including PV and ET (5–8). In the present study, the JAK2 gene mutation statuses of 130 patients and 10 controls from the Department of Pathology, Affiliated Hospital of Nanjing University of Traditional Chinese Medicine (Nanjing, China) were identified, and the corresponding clinical diagnosis was determined to investigate the value of JAK2 mutational analysis in MPN diagnosis. Scott *et al* (7) identified that JAK2 gene mutations commonly occurred on exon 12 in V617F-negative erythrocytosis patients. Thus, the patients in the present study were recommended to undergo exon 12 and 14 testing.

## Materials and methods

### Subjects

A total of 130 patients with hematological abnormalities (75 males and 55 females) and 10 healthy controls at the Affiliated Hospital of Nanjing University of Traditional Chinese Medicine were enrolled in the present study between November 2007 and March 2012. The mean patient age was 54.6 years (range, 11–85 years). A total of 80 patients exhibited MPNs [28 patients were diagnosed with PV (26 of which were clinically diagnosed), 32 with ET (27 of which were clinically diagnosed), 10 with PMF, two with chronic myeloid leukemia (CML) and 8 with other MPNs] and 50 exhibited non-MPNs [12 patients were diagnosed with leukocytosis, one with acute myeloid leukemia (AML), 10 with myelodysplastic syndrome (MDS), and 27 with other undiagnosed hematological abnormalities, such as leukocytosis, erythrocytosis and thrombocytosis] (Table I). In addition, the JAK2 mutation status of the 10 healthy individuals was investigated; this control group included six males and four females, with an average age of 56.9 years (range, 30–78 years). The patients were diagnosed according to the 2008 World Health Organization (WHO) classification (2).

### DNA extraction

Genomic DNA was isolated from peripheral blood leukocytes using the E.Z.N.A.^®^ Blood DNA and Tissue DNA kits (Omega Bio-Tek, Inc., Norcross, GA, USA). A spectrophotometer (Eppendorf BioPhotometer; Eppendorf, Hamburg, Germany) was used to determine the concentration of DNA, which was to be >2.0 μg/μl.

### Polymerase chain reaction (PCR) and purification

Primers for exon 12 and 14 were designed based on the JAK2 sequence obtained from the NCBI GenBank (http://www.ncbi.nlm.nih.gov/gene/3717) and were constructed by Sangon Biotech Co., Ltd. (Shanghai, China). The primers only generated a 203-bp product in the presence of the V617F mutation. PCR analyses (20 μl) were performed using 10X PCR buffer, 0.5 μl HotStarTaq DNA polymerase, 5X Q-Solution, 200 μM dNTP mix, 200 nM primer, DNA template (range, 200–300 ng; all from Qiagen, Hilden, Germany) and 8.8 μl distilled water. The PCR conditions were as follows: 95°C for 10 min, 45 cycles at 95°C for 15 sec, 54°C for 1 min and 60°C for 1 min, and a final extension at 72°C for 1 min. A PCR reaction without templates was performed as the negative control. All PCR products were analyzed by agarose gel electrophoresis and ethidium bromide staining. The PCR products were purified using the AxyPrep™ PCR Clean-Up kit (Axygen Biosciences, Union City, CA, USA).

### Sequencing

A cycle sequencing reaction was performed using 0.8 μl BigDye^®^ Terminator kit (version 3.1; Applied Biosystems Life Technologies, Foster City, CA, USA), 1.6 μl BigDye Sequencing Buffer (Applied Biosystems Life Technologies), 0.3 μl forward/reverse primer and 1 μl purified PCR product, adding double-distilled H_2_O to a total volume of 10 μl. The sequencing reaction conditions were as follows: 96°C for 1 min, followed by 30 cycles at 96°C for 10 sec, 50°C for 5 sec, 60°C for 4 min, and a final extension step at 60°C for 4 min. Electrophoresis of the purified sequencing product was performed using the ABI ABI PRISM^®^ 3100 Genetic Analyzer (Applied Biosystems Life Technologies) and the DNA sequence was analyzed using SeqScape software (version 2.1; Applied Biosystems Life Technologies).

### Statistical analysis

The χ^2^ test was used for group comparisons. P<0.05 was considered to indicate a statistically significant difference. All statistical analyses were performed using SPSS software, version 13.0 (SPSS, Inc., Chicago, IL, USA).

### Ethical approval

The present study was approved by the Ethical Committee of Nanjing University of Traditional Chinese Medicine and written informed consent was obtained from all participants.

## Results

**Figure 1:**
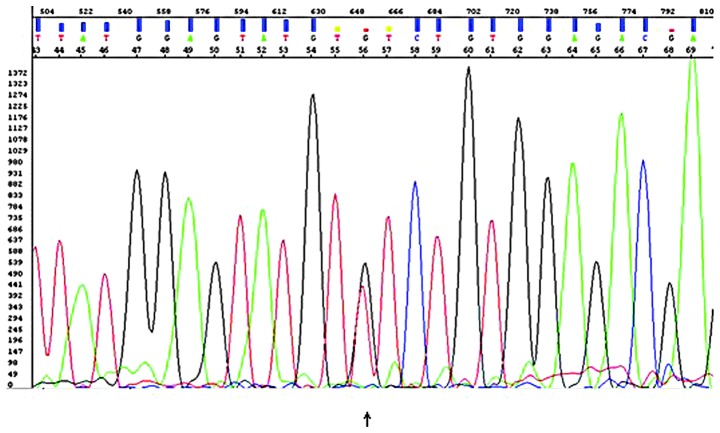
Representative sequencing data for the Janus kinase 2 V617F mutation from a clinically diagnosed polycythemia vera patient.

All 140 patient samples were analyzed for the JAK2 mutation using direct sequencing. Among them, 74 samples were tested for exons 12 and 14 (36 MPNs, 31 non-MPNs and 7 controls), while 66 samples were tested for exon 14 only (44 MPNs, 19 non-MPNs, three controls); this was predominantly due to economic considerations, as patients were required to fund their own tests As all of the participants undertook exon 14 testing and no mutation was identified in exon 12, the results for the exon 14 test only were analyzed (Table I). The mutation rate of the total samples was 41.5% (54/130) and all of the samples were heterozygous, however, no JAK2 V617F mutations were identified in the 10 controls. Of the 26 patients with clinically diagnosed PV, 21 (80.8%) exhibited the V617F mutation (Fig. 1). The JAK2 V617F mutation was also identified in two patients with clinically suspected PV, who exhibited erythrocytosis but did not satisfy the clinical criteria for PV. When using the JAK2 V617F mutation as a supplemental criterion for the diagnosis of PV, as described by the WHO (2), two patients with clinically suspected PV prior to JAK2 testing were diagnosed and added to the PV group; the adjusted mutation rate was 82.1% (23/28). Similarly, the mutation rate of V617F and other mutations in ET was 44.4% (12/27), while the adjusted mutation rate was 53.1% (17/32), as five patients with clinically suspected ET were added to the ET group following JAK2 testing. The JAK2 mutation rates in PMF and other MPNs, including two CML samples, were 40.0% (4/10) and 60.0% (6/10), respectively. The two CML samples and 4/12 patients (33.3%) with leukocytosis of unknown origin were identified to exhibit the V617F mutation. As a result, the adjusted total mutation rate of the JAK2 gene in MPN cases was 62.5% (50/80). Furthermore, the present study identified a synonymous mutation of Y626Y in one patient with PV and a C/T point mutation in intron 14 of one patient with ET.

The V617F mutation was only identified in 4/12 patients with leukocytosis of unknown origin (33.3%); however, the mutation was not identified in any other non-MPN samples, including AML (0/1), MDS (0/10) and others (0/16). Therefore, a significant difference was identified between the rate of JAK2 mutation in MPN and non-MPN patients (P=0.000). Additionally, the JAK2 mutation rate in MPN patients was significantly different from that in the control group (P=0.000).

## Discussion

MPNs are a group of clonal hematopoietic stem cell malignancies characterized by the excessive production of one or more cell types in the bone marrow (for example, myeloid, erythroid megakaryocyte or mast cells), but usually with no maturity. The clinical manifestations are heterogeneous and fibrotic, and leukemic transformation often occurs later. Numerous studies have reported that patients with MPN carry a novel molecular genetic abnormality, the JAK2 V617F point mutation. Additionally, these previous studies indicate that the V617F mutation is the major molecular maker for pathogenesis in BCR/ABL-negative MPN patients, therefore, the V617F mutation may act as an important molecular genetic and diagnostic marker for BCR/ABL-negative MPN (5,6,9,10). The V617F mutation occurs in hematopoietic stem cells and confers proliferative and survival advantages to hematopoietic precursors. Recently, the diagnostic criteria for PV, ET and PMF were revised, to incorporate the described molecular markers (JAK2 mutation) (2). In the present study, the V617F mutation rate of PV was 80.8% (21/26). When using JAK2 V617F mutation as a supplemental criterion to diagnose PV, the adjusted mutation rate was 82.1% (23/28). Similarly, the mutation rate of ET increased from 44.4% (12/27) to 53.1% (17/32), using the supplemental criteria. Evidently, the JAK2 V617 mutation detection may be an important method for hematologists to diagnose of MPN.

Typically, the V617F mutation has been investigated in the peripheral blood (5,6). In the present study, genomic DNA was isolated from peripheral blood leukocytes or directly from the bone marrow. The presence of the JAK2 mutation at exons 12 and 14 was investigated in 130 patients by direct sequencing. No mutations were identified in exon 12, a result that is inconsistent with a previous study, which reported a high percentage of JAK2 exon 12 mutations in V617F-negative PV patients of Asian origin (11). As the Taiwanese and mainland Chinese share the same genetic background, this inconsistency in exon 12 mutation rates cannot be explained by geographic differences. Thus, additional data from the general population of Asia are required when conducting future studies.

Although the JAK2 V617F mutation has been frequently identified in BCR/ABL-negative MPN and is rarely present in other hematologic disorders, this mutation does occur in other hematologic malignancies, such as refractory anemia with ring sideroblast, AML and systemic mastocytosis (12,13). Despite this, the JAK2 V617F mutation may still be valuable in the diagnosis of MPN; for example, a previous study indicated that JAK2 V617F allele evaluation may be used to discriminate MDS from MPN in specimens with bone marrow fibrosis (14).

In the present study, the total positive mutation rate was 41.5% (54/130) including, 82.1% in PV, 53.1% in ET and 40.0% in PMF, which was consistent with the scientific literature (ranges: PV, 65–97%; ET, 23–57%; PMF, 35–57%) (3,4,5,6). These results revealed that the positive rate of JAK2 V617F mutation in MPN (62.5%) is significantly different from that in non-MPN (8.0%); therefore, the incidence of the JAK2 V617F mutation may be a reliable diagnostic criterion in MPN. Notably, a synonymous mutation of Y626Y was identified in one patient with PV and a C/T point mutation was identified in intron 14 of an ET patient. It remains unclear whether these were coincidental findings, genetic markers for Asian individuals or a reflection of genomic instability. Additional data is required to determine the reasons for these non-V617F mutations.

The role of the JAK2 V617F mutation in the mechanism of MPN subtype development remains to be investigated and may result in novel studies regarding target agents (15). Direct sequencing is an important method for JAK2 mutation screening as the accuracy and repeatability of the process meets the requirements for clinical testing and provides support during clinical diagnosis.

## Figures and Tables

**Table I:** JAK2 V617F mutation rate on exon 14 in each group (n=140).

Disease	Cases, n	V617F mutation, n	Other mutation	Mutation rate, %
MPN				
PV	28	23	Y626Y	82.1^a^
CD PV	26	21		80.8
CS PV	2	2	None	100.0
ET	32	17	Intron 14 C/T	53.1^a^
CD ET	27	12		44.4
CS ET	5	5	None	100.0
PMF	10	4	None	40.0
CML	2	2	None	100.0
Other MPN	8	4	None	50.0
Non-MPN				
Leukocytosis of unknown origin	12	4	None	33.3
AML	1	0	None	0.0
MDS	10	0	None	0.0
Others	27	0	None	0.0
Control	10	0	None	0.0

a Modified mutation rate, based on the JAK2 mutation test result.

JAK, Janus kinase; MPN, myeloproliferative neoplasms; PV, polycythemia vera; CD, clinically diagnosed; CS, clinically suspected; ET, essential thrombocythemia histiocytosis; PMF, primary myelofibrosis; CML, chronic myeloid leukemia; AML, acute myeloid leukemia; MDS, myelodysplastic syndrome.
